# Human macrophages release exosomes containing anti-inflammatory microRNAs after phagocytosis of *Leishmania infantum*

**DOI:** 10.3389/fimmu.2025.1593829

**Published:** 2025-12-10

**Authors:** Cinthia L. Hudachek, Bayan Zhanbolat, Yani Chen, Mary E. Wilson

**Affiliations:** 1 Department of Microbiology and Immunology, Carver College of Medicine, University of Iowa, Iowa City, IA, United States; 2 Department of Internal Medicine, Carver College of Medicine, University of Iowa, Iowa City, IA, United States; 3 Veterans’ Affairs Medical Center, Iowa City, IA, United States

**Keywords:** Leishmania infantum, visceral leishmaniasis, exosomes, microRNAs, human macrophage

## Abstract

**Introduction:**

The protozoan *Leishmania infantum* causes visceral leishmaniasis, a disease associated with suppressed systemic innate and adaptive immune responses. Mechanisms underlying the generalized immune suppression are incompletely understood. Exosomes are a subset of microvesicles released from eukaryotic cells, which contain proteins, lipids, and nucleic acids, including microRNAs (miRNAs). These small regulatory RNAs can simultaneously modify the expression of many genes and pathways. We hypothesized that *L. infantum* infection of macrophages induces the release of exosomes containing immunomodulatory miRNAs that contribute to systemic immunosuppression during visceral leishmaniasis.

**Methods:**

Using NanoString arrays, we profiled exosomal miRNAs released by infected versus uninfected human macrophages. Differential expression was validated by RT-qPCR, and functional effects were tested by transfecting miR-mimics.

**Results:**

Several miRNAs were augmented in infected exosomes, including hsa-miR-223-3p which has anti-inflammatory activities. Previous studies showed that the NLRP3 inflammasome pathway might be targeted by differentially expressed miRNAs, a hypothesis that was confirmed in transfected THP-1 monocytic cells overexpressing hsa-miR-223-3p.

**Discussion:**

We show that *L. infantum* infection induces the release of exosomes containing miRNAs that are capable of modifying the host immune environment, possibly suppressing microbicidal pathways and favoring parasite survival.

## 1 Introduction

Visceral Leishmaniasis (VL) is a chronic, systemic parasitic infection, usually caused by *Leishmania infantum* in the western hemisphere and either *L. infantum* or *L. donovani* in the eastern hemisphere (1). Infection with *L. infantum* can remain asymptomatic for years or it can cause symptomatic VL, accompanied by suppression of systemic immunity with failure of adaptive T cell responses (2, 3). Clinical signs of VL are fever, weight loss, hepatosplenomegaly, anemia, leukopenia, and suppression of both parasite-specific and generalized immune responses (4). As a result of widespread immunosuppression, death often occurs due to secondary bacterial infection (5). During chronic infection with the visceralizing *Leishmania* species, disseminated parasites establish intracellular infection and reside long-term in tissue-resident macrophages of reticuloendothelial organs, including the spleen or liver (1). The macrophage can serve either as a quiescent home for the parasite, or an intracellular niche enabling parasites to cause systemic disease.

Exosomes are a subset of extracellular microvesicles (EVs) that are released from most or all eukaryotic cells through budding from the lumen of the multivesicular body. Exosomes are released from both the parasite and the infected host cell (6, 7). They contain nucleic acids (microRNA, messenger RNA, DNA), lipids, and an array of proteins including cytokines, surface receptors and proteases, and lipids (8). Exosomes can be found in the bloodstream, secretions, and urine of mammals (9). These small vesicles are thought to communicate between cells in distant tissues by delivering cytokines, proteases, and other modulating molecules between cells without direct cell-cell contact (6, 10–12). Exosomes have been shown to modulate immune responses in leishmaniasis both systematically *in vivo* (13) and for individual cell types *in vitro* (14). Although it is uncertain which of the cargo carried by exosomes cause these biological effects, microRNAs (miRNAs) are attractive candidates since they can potentially exert widespread effects on immune response genes (15). miRNAs are endogenous 18–25 nt non-coding RNAs that simultaneously regulate the expression of genes by either inhibiting translation or degrading target transcripts, through base-pairing between a 6–8 nt seed sequence in the miRNA to a complementary or near-complementary sequence in the mRNA, often in the 3’- or the 5’-UTR (16). microRNAs have been shown to contribute to immune responses to both cancer and infection (17–19).

Because VL is associated with systemic suppression of host responses (5), and because exosomes containing miRNAs are distributed widely through the bloodstream (9, 10, 15), we hypothesized that the miRNA content of exosomes released from macrophages would be modified by infection with *L. infantum*. Differences in miRNA content might in turn modify the immune response during infection. We investigated our hypothesis by screening all known human miRNAs, using a miRNA profiling assay from NanoString to compare miRNAs in extracellular vesicles released from infected versus uninfected human macrophages. Our data revealed that infection caused significant changes in the profile of miRNAs released from macrophage exosomes, some of which have been associated with the suppression of host antimicrobial responses. These findings may help illuminate a cellular mechanism contributing to the immune landscape during VL.

## 2 Materials and methods

### 2.1 Parasites

A Brazilian strain of *L. infantum* (MHOM/BR/00/1669) was maintained by serial passage in Syrian hamsters. Promastigotes were maintained in liquid culture in hemoflagellate-modified minimal essential medium (HOMEM) (20) and used within 3 weeks of isolation.

### 2.2 Isolation of peripheral blood mononuclear cells

Peripheral blood leukocytes were extracted from Leukocyte Reduction System (LRS) cones from healthy blood donors to the University of Iowa DeGowin Blood Donation Center (21). Blood donors had no underlying chronic diseases and were not taking immunosuppressive medications. Peripheral blood mononuclear cells (PBMCs) were isolated from the LRS cones by density on Ficoll-Paque™ PLUS density gradient (Cytiva) according to the manufacturer’s instructions. PBMCs were incubated in 10cm cell culture dishes in Advanced RPMI 1640 Medium (Gibco) supplemented with 1% penicillin/streptomycin (Gibco) and 10% v/v heat-inactivated exosome depleted fetal bovine serum (BioWest) supplemented with 5 ng/mL human Macrophage colony-stimulating factor (Millipore Sigma) and incubated at 37oC, 5% CO2. Exosome depleted FBS was generated by excluding microvesicles with a 10 kDa cutoff Amicon ultrafiltration tube (Millipore Sigma) for 30 min at 2,095 x g at 4 °C (22). Mononuclear phagocytes were isolated from PMBCs by adherence to tissue culture dishes overnight, after which non-adherent cells were removed by rinsing.

### 2.3 Infection of monocytes

Promastigotes in stationary phase of growth were pelleted in Hanks Balanced Salts Solution (HBSS) and opsonized by incubation in 5% pooled human serum/HBSS for 30 minutes at 37°C. Promastigotes were washed by centrifugation. Mononuclear phagocytes were infected with opsonized promastigotes at a 40:1 parasite: mononuclear phagocyte ratio. Sterile coverslips were included in some culture wells to assess infection efficiency microscopically. Infections were synchronized by centrifugation at 21 x g for 5 mins without brake. After 16 hrs at 37°C, 5% CO_2_, parasites were removed by rinsing 7x times with HBSS and fresh Advanced RP10 was added. Culture plates were returned to 37°C, 5% CO_2_ for a total of 4 days of infection. Media was changed after 48 hrs and supernatants were collected for exosome isolation after an additional 48 hrs since the last change of exosome-depleted media.

### 2.4 Exosome isolation

Cell supernatants were passed through a 0.22µm filter (Millipore Express^®^ PLUS Membrane, Millipore Sigma) to remove intact cells and large fragments. The supernatants were concentrated by sequential centrifugation in Amicon^®^ Ultra Centrifugal Filter10kDa cutoff filtration tubes (Millipore Sigma) (2,095 x g at 4 °C) until reduced to 1ml. EVs were isolated by ultracentrifugation at 110,000 x g for 1 hour, and pellets were rinsed gently with 4°C PBS. EVs were resuspended in PBS and stored at -20 °C. The purity of preparations was assessed by NanoParticle tracking analysis (see below).

### 2.5 Nanoparticle tracking analysis

The purity and quality of each extracellular vesicle preparation was assessed using nanoparticle tracking analysis as described (23). The size and uniformity of EVs were assessed on the ViewSizer3000 (Horiba Scientific Technologies). Ten percent of the total exosome isolation was aliquoted for nanoparticle tracking analysis (NTA) in HBSS. Five hundred µls were added to a cuvette with a stir bar and inserted into the ViewSizer3000. The ideal diluted sample has 20–100 visible particles on the screen. The ViewSizer3000 stirred the sample for 5 seconds, followed by a 3 second rest before recording a 10 second video. This process was repeated a total of 30 times for 30 videos per sample. The machine tracks the size and concentration of nanoparticles captured during the 10 second videos with 445 nm, 520 nm, and 635 nm lasers.

### 2.6 RNA isolation

Exosomes, MDMs, or THP-1 cells were suspended in 1ml of TRIzol (InVitrogen) and RNA was isolated according to the manufacturer’s protocol followed by overnight precipitation at -80 °C in 0.1 vols of 3M sodium acetate, 3 volumes of ice-cold 100% ethanol, and 3 µl 5 mg/ml linear acrylamide (Thermo Fisher Scientific, Waltham, MA). RNA was pelleted (17,950 xg for 30 mins at 4°C), rinsed in 75% ice cold EtOH and treated with DNase (Zymo RNA Clean & Concentrator-5, Zymo Research Corp.) according to the manufacturer’s instructions. The final RNA concentration was analyzed using a NanoDrop spectrophotometer model ND-1000 (ThermoFisher).

### 2.7 NanoString miRNA assay

Exosome RNA samples were screened with the NanoString nCounter Human V3 miRNA panel (NanoString, Seattle, WA) to detect the profile of miRNAs contained within exosomes of primary human macrophages infected with *L. infantum* or uninfected controls. The nCounter panel detects the 827 known human miRNAs with specific capture and bar-coded reporter probe pairs. 100 ng of total RNA for each sample were analyzed using the manufacturer’s instructions (NanoString nCounter miRNA Expression Assay User Manual MAN-C0009-08). Probe hybridization measured in fluorescence intensity was used to quantify miRNAs present in each sample. The limits of detection were determined with positive and negative control samples. This resulted in raw hybridization counts, which were normalized to the top 100 expressed miRNAs using the nSolver analysis software (NanoString, Seattle, WA).

### 2.8 RT-qPCR validation of miRNA and mRNA expression levels

cDNA was generated from total RNA using TaqMan miRNA Assays and TaqMan™ MicroRNA Reverse Transcription Kit according to the manufacturer’s instructions. TaqMan assays, master mix and kits were from ThermoFisher). *hsa-miR-223-3p, hsa-miR-23a-3p, hsa-miR-575, hsa-miR-1285-5p*, and *hsa-miR-1305* TaqMan miRNA Assays were used to significant differences. *hsa-miR-16-5p* was chosen as an internal control due to its stable expression in our and others’ assays (24). Fold changes were calculated using the 2^-ΔΔCT^ method.

Gene expression was evaluated using RT-qPCR. cDNA was generated with SuperScript™ III First-Strand Synthesis. TaqMan assays for specific mRNA, and TaqMan™ Fast Advanced Master Mix were obtained from Thermo Fisher. Fold changes in gene expression were calculated using the 2^-ΔΔCT^ method, normalizing to the housekeeping gene *ACTB*.

### 2.9 Gene target prediction and pathway analysis

The predicted mRNA targets of differentially expressed miRNAs that RT-qPCR validated were assessed using the bioinformatic prediction programs miRDB (https://mirdb.org/) and TargetScan (https://www.targetscan.org/vert_80/). Targeted genes were assigned Gene Ontology terms and analyzed for common pathways and functions using the Gene List Analysis program of PANTHER Classification System version 19.0 (www.pantherdb.org). Tables were imported into R and analyzed to determine genes that overlapped between potential targets of different miRNAs.

### 2.10 Transfection of THP-1 cells with miRNA mimics

The THP-1 human monocytic cell line was transfected with either mirVana™ miRNA Mimic Negative Control #1 (scramble) or mirVana™ miRNA Mimic *hsa-miR-223-5p* (ThermoFisher) using Lipofectamine™ RNAiMAX Transfection Reagent (InVitrogen). THP-1 cells were cultured in RP10 medium (RPMI, 100 U/mL penicillin/streptomycin, 10% heat-inactivated fetal calf serum). THP-1 cells at 2 x 10^5^ per well in complete RPMI were seeded in 24-well plates. Three µl of Lipofectamine™ RNAiMAX Transfection Reagent and 10 pmol of miRNA mimics were combined in 100 µl of Opti-MEM™ I Reduced Serum Medium, incubated at room temperature for 15 mins, and added to cells. Transfection reactions were incubated at 37°C, 5% CO_2_. Twenty four hours later, THP-1 cells were induced to differentiate toward macrophages with the addition of 80 ng/ml Phorbol 12-myristate 13-acetate (PMA) (Tocris) and cultured in RP-10 for 3 days at 37°C (25). THP-1 cells were then washed with PR10 and were left to rest in RP-10 for 3 days at 37°C before infection.

THP-1 cells were infected with opsonized *L. infantum* promastigotes and RNA was collected as described above. RNA was collected after 24 hours of infection.

### 2.11 Quantification and statistical analyses

Statistical analyses of normalized miRNA counts were performed using Prism GraphPad software. NTA data was analyzed using a ratio paired t-test. NanoString data and transfection data were analyzed with two-way analysis of variance (ANOVA) followed by Dunn’s multiple comparisons test. RT-qPCR comparisons of miRNA were performed with paired t-test. Corrected p-values of <0.05 were considered significant.

## 3 Results

### 3.1 *L infantum* infection increases the number of released exosomes

**Figure 1:**
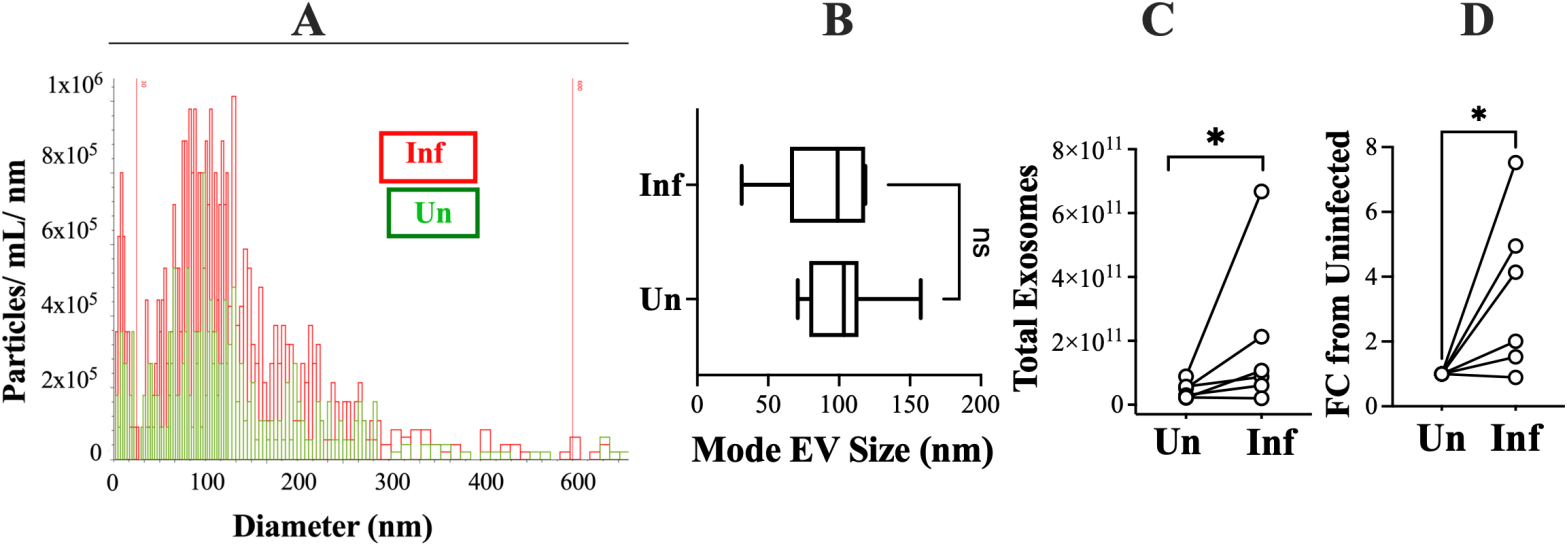
Quantification and size analysis of EVs released from macrophages with or without *L. infantum* infection. EVs were isolated via ultracentrifugation analyzed for size distribution and concentration by 3-laser diffraction using the View Sizer 3000. **(A)** Shown in this panel is a representative nanoparticle tracker graph of EVs released from one set of uninfected cells (Un, green) and infected cells (Inf, red) from a single individual. **(B)** Using macrophages from six healthy donors, we measured the size and numbers of EVs released using the nanoparticle tracking method illustrated in panel **(A)** Measurements represent the average between two 30-second videos for each sample. The graph shows a box and whisker plot with lower 25^th^ percentile, median and upper 75^th^ percentile in the box, and whiskers from minimum to maximum values. **(C)** As in panel **(B)** nanoparticle tracker data were used to compare the total concentration of EVs released from uninfected versus infected cells. **(D)** The data in **(C)** are expressed as the fold-change in EV numbers release from infected cells normalized to uninfected cells from the same individual. Statistical comparisons were done using a paired t-test **(B, C)** or a ratio-paired t-Test **(C)**. Inf: exosomes released from infected cells; Un: exosomes released from uninfected cells. *p<0.05.

Human monocyte-derived macrophages (MDMs) were generated from peripheral blood leukocytes of six healthy donors from the University of Iowa DeGowin Blood Donation Center. EVs were collected from MDMs that were either uninfected or infected with *L. infantum.* The purity, size, distribution, and number of particles were assessed by nanotracker analysis, using 3-laser diffraction with a ViewSizer 3000 (Horiba Scientific). An example of such a scan is shown in **Figure 1A**, and collated measurements from nanoparticle tracker analyses of macrophage EVs from all six blood donors are shown in **Figures 1B–D**. Similar to prior reports, the majority of EVs conformed to the uniformity of size and distribution of exosomes (26) (**Figure 1**). Because of this, we will refer to the EVs as exosomes in this study. The exosomes released from paired infected or uninfected cells were analyzed. There was no difference in exosome size between infected versus uninfected cells. Nonetheless there was an increase in exosomes released from MDMs in response to infection, when quantified either as number of exosomes (p=0.0279) or fold change exosome released from infected compared to uninfected cells (p=0.0287) (**Figure 1**).

### 3.2 Exosomes from infected and uninfected cells contained different miRNA profiles

A screen of human miRNAs was performed to determine whether *L. infantum* infection changes the profile of miRNAs released by human MDMs. We used the NanoString nCounter Human V3 miRNA panel, which assays the expression of 827 known human miRNAs. After internal quality assessment, miRNA probe hybridization counts were normalized to the top 100 expressed miRNAs. Counts above the threshold of 20 were considered to have expression above the background levels. The original nanoString counts, excluding controls, are included in **Supplementary Table 1**.

**Figure 2:**
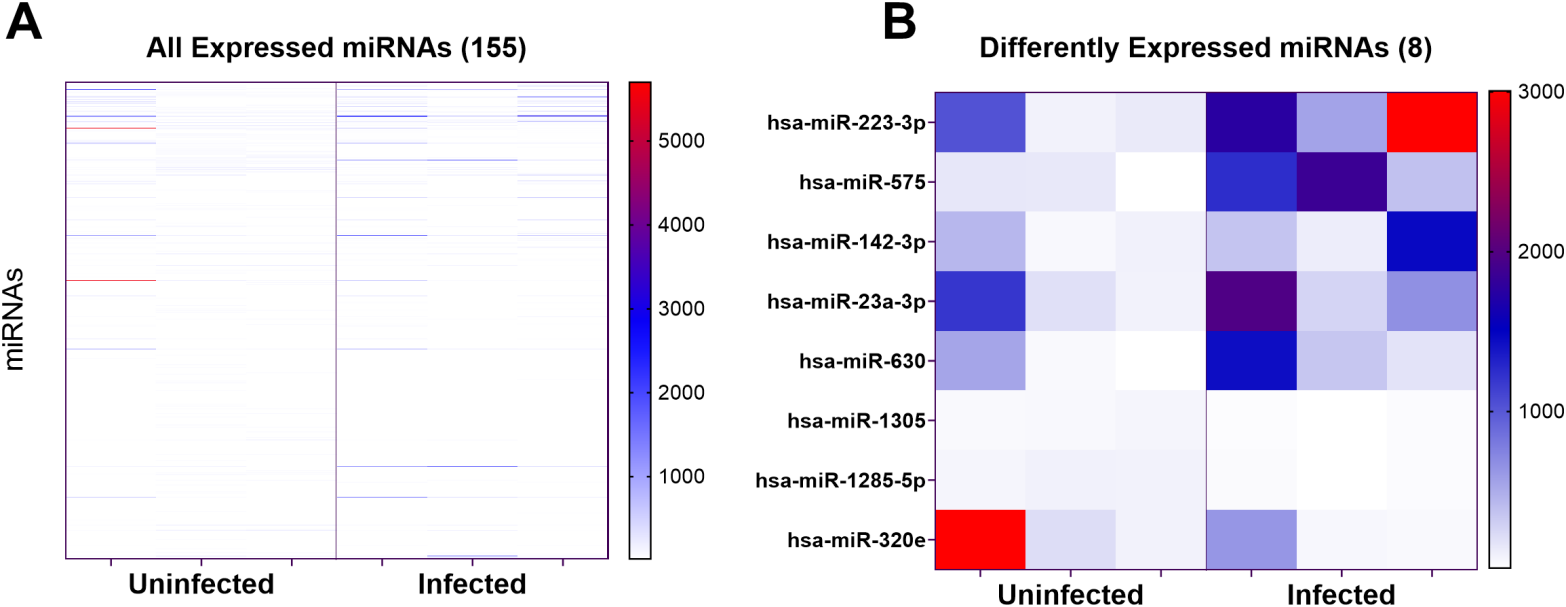
Differentially expressed miRNAs from exosomes of macrophages infected with *L. infantum* or uninfected controls. RNA was isolated from isolated exosomes. miRNAs were normalized to the top 100 expressed miRNAs. The value scale on the Y-axis represents normalized miRNA counts. **(A)** A heat map of all expressed human miRNAs in the NanoString assay is shown. **(B)** A heatmap was generated to illustrate the subset of miRNAs that were shown to be differ significantly between exosomes released from infected macrophages and those released from uninfected macrophages, when analyzed via two-way ANOVA or multiple t-tests followed by FDR. The scale map illustrates normalized miRNA counts. Please note that the most highly upregulated miRNAs are indicated with red. miRNAs at the basal state are blue, and the highly down-regulated miRNAs are white.

Among the 827 screened, a total of 155 miRNAs were expressed in at least one subject from one of the phenotype groups (**Figure 2A**). After correction for multiple testing, eight miRNAs differed significantly between exosomes from uninfected and infected MDMs. These included *hsa-miR-223-3p*, *hsa-miR-575, hsa-miR-23a-3p, hsa-miR-142-3p, and hsa-miR-630* which were increased in exosomes from infected cells, and *hsa-miR-1285-5p*, *hsa-miR-1305*, and *hsa-miR-320e* which were decreased in exosomes from infected cells (**Figure 2B**; **Table 1**).

**Table 1:** Significantly different miRNAs.

miRNA	Fluorescence units (counts)		Fold- change	p adj FDR	p adj Tukey’s test
	Average uninfected	Average infected			
hsa-miR-1285-5p	90.2500	34.1233	0.3781	***0.0045***	>0.9999
hsa-miR-1305	61.9733	31.4900	0.5081	***0.0219***	>0.9999
hsa-miR-320e	1936.7900	248.1067	0.1281	0.3990	***<0.0001***
hsa-miR-575	110.2600	1160.0733	10.5213	0.0710	***<0.0001***
hsa-miR-223-3p	421.5100	1769.1867	4.1973	0.1553	***<0.0001***
hsa-miR-23a-3p	495.1767	968.4400	1.9557	0.4895	***<0.0001***
hsa-miR-630	207.2300	654.5233	3.1584	0.3540	***<0.0001***
hsa-miR-142-3p	197.3367	650.3200	3.2955	0.3514	***<0.0001***

All miRNAs shown to be significantly different between uninfected and infected are listed. These correspond to miRNAs illustrated in the heatmap in **Figure 2B**. The data show normalized expression of each miRNA measured in fluorescence units, and comparisons of the fold change in infected versus uninfected MDMs. Statistical comparisons of normalized fluorescence units were done using both a 2-way ANOVA followed by Tukey’s correction for multiple comparisons (p adj Tukey’s Test), and by comparing the fold changes with multiple t-tests followed by Benjamini Hochberg FDR correction for multiple testing (p adj FDR). miRNAs that differed significantly between conditions by either criterion are included in this table and in **Figure 2B**.Bold font indicates statistically significant adjusted p values.

**Figure 3:**
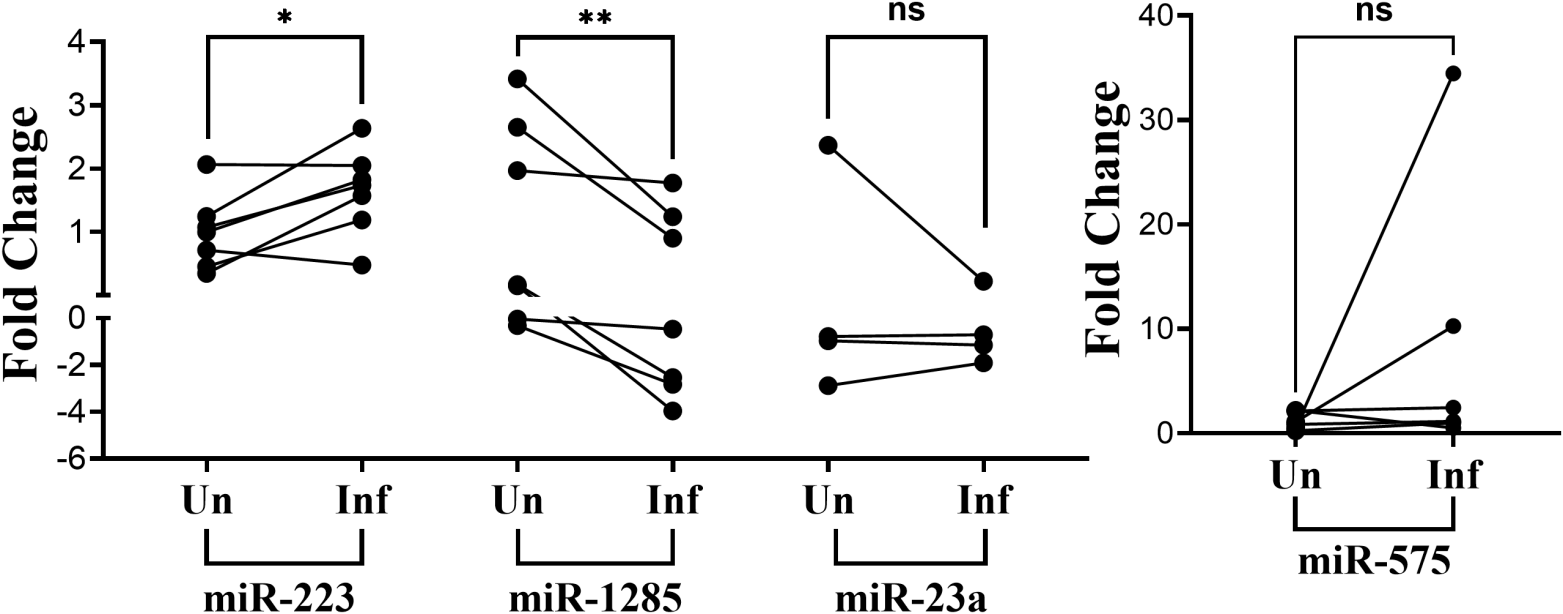
RT-qPCR validation of differentially expressed miRNAs. RNA was isolated from exosomes released by infected versus uninfected human MDMs. Five target miRNAs and one normalizing (constant) miRNA (miR-16) were amplified from reverse transcribed RNA. Raw Ct values were normalized to the mean CT of the normalizing miRNA (hsa-miR-16). The 2^-ΔΔCt^ method was used to calculate fold differences from the mean delta CT of uninfected macrophages. A paired t-test was used to establish significance. Data for the fifth candidate, *hsa-miR-1305*, are not shown because Ct values were below the detection threshold. *p<0.05, **p<0.01, ns, not significant.

Five miRNAs that differed significantly between the groups according to the NanoString assay were validated by reverse transcriptase-qPCR using miRNA Taqman assays. These miRNAs (*hsa-miR-223-3p, hsa-575*, *hsa-miR-23a-3p, hsa-miR-1285-5p, hsa-miR-1305)* were chosen because they had the highest fold-increase or -decrease in infected compared to uninfected MDM exosomes. While not one of the most increased or decreased miRNA in exosomes from the infected macrophages, *hsa-miR-23a-3p, hsa-miR-1285-5p, and hsa-miR-1305* were also chosen for validation because they were also increased in a previous study from our lab that documented miRNAs which were increased in plasma of patients with visceral leishmaniasis compared to endemic controls (in press, Roy, Hudachek, Wilson et al.). RT-qPCR was performed with exosomes from the seven donors contributing samples for size and density analyses in **Figure 1**. *hsa-miR-223-3p* (p=0.0271) was again found to be significantly increased via RT-qPCR and *hsa-miR-1285-5p* (p=0.0082) was significantly decreased (**Figure 3**). However, expression of *hsa-miR-575* (p=0.3) or *hsa-miR-23a-3p* (p=0.875) did not differ significantly between exosomes from infected versus uninfected macrophages (**Figure 3**). Ct values for *hsa-miR-1305* were below the detection level in all samples, indicating the expression of this miRNA was very low.

### 3.3 Target prediction and pathway analysis prediction

miRNAs released from mammalian cells will often contribute synergistically to modify pathways, which may be easier to detect than changes in expression of individual genes. The two miRNAs shown on validation assays to be significantly differentially expressed in exosomes from infected compared to uninfected cells were *hsa-miR-223-3p* (increased) and *hsa-miR-1285-5p* (decreased). We generated a list of predicted targets of these two miRNAs using miRDB (Version 6.0, https://mirdb.org/) and TargetScan Human (Release 8.0, https://www.targetscan.org/vert_80), listed in **Supplementary Table S2**. A total of 228 genes were. predicted targets of the two miRNAs, indicated in column K of the table.

**Figure 4:**
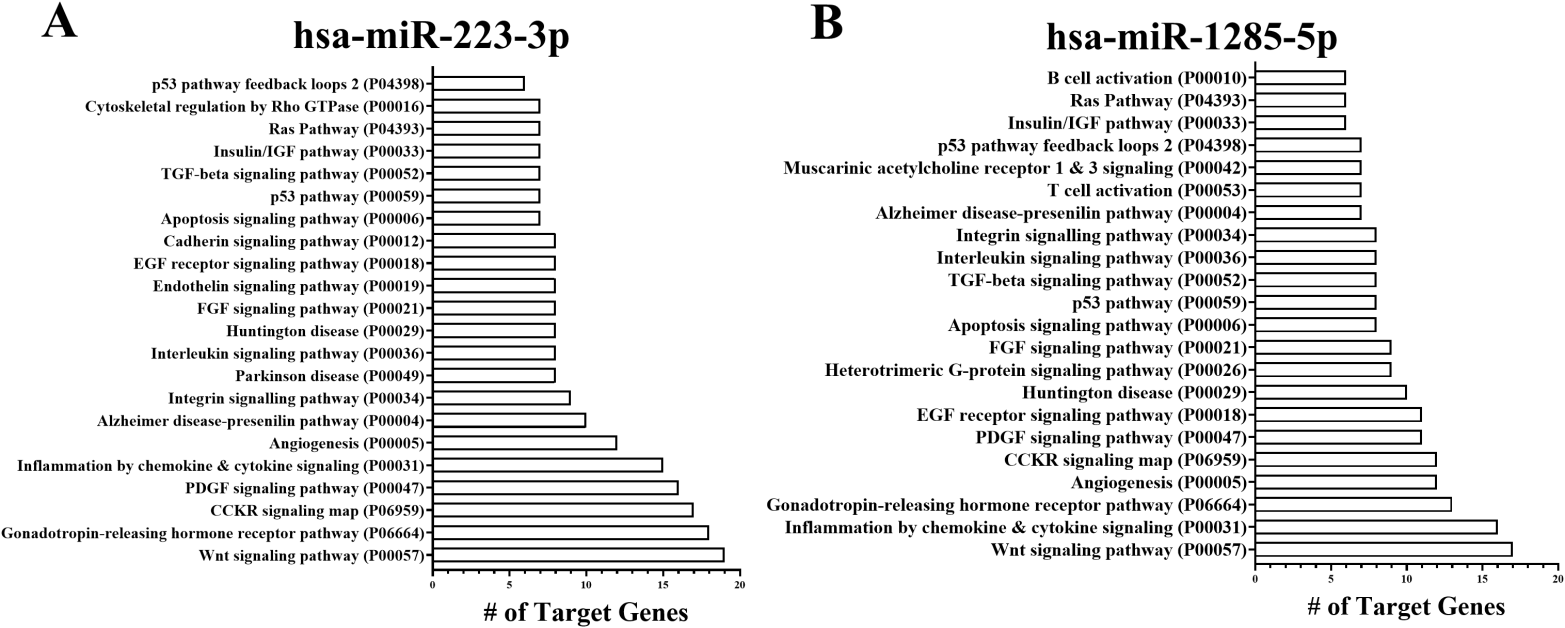
Pathway analysis of hsa-miR-223-3p and hsa-miR-1285-5p predicted gene targets. Target mRNAs for each of the two validated miRNAs were generated using miRDB and TargetScan. These were concatenated and uploaded into the Panther Gene Ontology pathway analysis program. Pathways that were significantly represented in these lists are shown in barplots for **(A)***hsa-miR-223-3p* and **(B)***hsa-miR-1285-5p*.

As the list was long, the predicted target genes for each miRNA were further analyzed using the Panther Pathway Analysis Program, which is based on Gene Ontology in the Panther suite of programs (Version 18.0, www.pantherdb.org). Data are summarized in **Figure 4**. “Inflammation mediated by chemokine and cytokine signaling” (P00031) was one of the top pathways represented among the predicted target pathways for both *hsa-miR-223-3p* and *hsa-miR-1285-5p* (**Figure 4**). Other top pathways shared by both lists included “Gonadotropin-releasing hormone receptor pathway” (P06664) and “Wnt signaling pathway” (P00057) (**Figure 4**). However it must be noted that infection led to up-regulation of *hsa-miR-223-3p* but significant down-regulation of *hsa-miR-1285-5p* (See **Figure 3**). Thus they would be predicted to have opposing effects on the same target genes. Thus it may not be surprising that study of the miRNA predicted targets contributing to the “Inflammation mediated by chemokine and cytokine signaling pathway” for each miRNA revealed only two predicted gene targets in common, PKCS1 and SOCS5 (**Supplementary Table S2**). For the above reason, we viewed it wisest to proceed with validation of targets of individual miRNAs.

### 3.4 *hsa-miR-223-3p* transfected cells result in a change in NLRP3 gene expression

**Figure 5:**
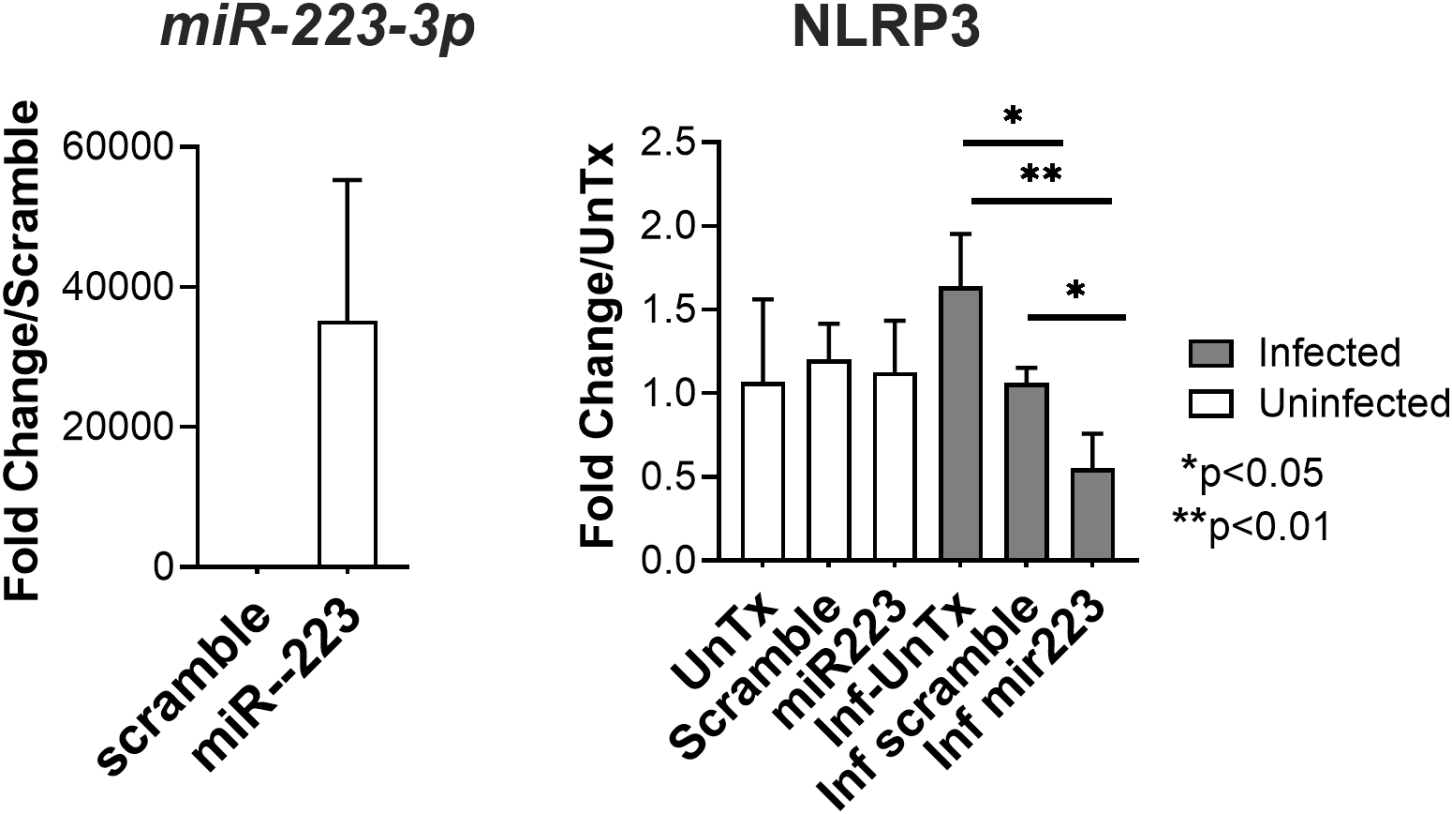
Gene expression in THP-1 Cells transfected with *hsa-miR-223-3p*. The monocytic cell line THP-1 was transfected with an *hsa-miR-223-3p* mimic or a scramble control, and then infected with *L. infantum*. Twenty-four hours later, expression of *hsa-miR-223-3p* and the putative target NLRP3 were assessed by RT-qPCR. Expression was normalized to the constant miRNA, hsa-miR-16. The 2 ^-ΔΔct^ method was used to calculate fold differences between transfected and scramble control (left panel) or between conditions and the mean of uninfected un-transfected (UnTx) wells. Data show the mean of three replicates. An unpaired t-test was used to assess significance (*p<0.05, **p<0.01).

*hsa-miR-223-3p* has been implicated in the immune pathophysiologic response to many bacterial and viral pathogens (27). Mechanisms implicated include its ability to suppress inflammatory responses of neutrophils, macrophages and dendritic cells, such as inhibiting inflammasome activation (28–32). Furthermore, our lab has shown that *hsa-miR-223-3p* is the most abundant up-regulated miRNA in the plasma of Indian patients with visceral leishmaniasis (in press, Roy, Hudachek, Wilson et al.). Since *hsa-miR-223-3p* was also the highest up-regulated miRNA in infected MDMs, we investigated its effects on gene expression in macrophages infected with *L. infantum.* The human monocytic cell line THP-1 (25, 33) was transfected with MirVana *hsa-miR-223-3p* mimic or a scrambled mimic as a negative control. Transfected cells were differentiated toward macrophages and incubated with *L. infantum* to allow phagocytosis (25). Twenty-four hours post-infection, RNA was collected and analyzed by RT-qPCR. The results documented an increase in *hsa-miR-223-3p* expression in THP-1 cells transfected with the *hsa-miR-223-3p* mimic compared to the negative control (**Figure 5**).

Among the predicted gene targets of *hsa-miR-223-3p* but not of *hsa-miR-1285-5p*, is NLRP3. Furthermore, *hsa-miR-223-3p* has been shown experimentally to target the *NLRP3* inflammasome pathway (34). We therefore assessed NLRP3 expression in *miR-223* overexpressing and control cells. THP-1 cells transfected with the *hsa-miR-223-3p* mimic expressed significantly lower levels of *NLRP3* mRNA compared to the scrambled miR-mimic control (p=0.041) (**Figure 5**). There was no difference seen in the expression of *NLRP3* between THP-1 cells transfected with the *hsa-miR-223-3p* mimic or the scramble control for the uninfected group (data not shown). Thus, *hsa*-miR-223-3p led to a decrease in NLRP3 expression that was induced by parasite infection, but not in the basal (un-induced) NLRP3 expression.

## 4 Discussion

VL is a potentially fatal outcome of infection that is caused in many parts of the world by the intracellular protozoan *Leishmania infantum*. Although infection is often asymptomatic, symptomatic VL is accompanied by progressive suppression of systemic adaptive immune responses (35, 36). The current study was based on the hypothesis that exosomes released from macrophages infected with *L. infantum* would contain miRNAs that influence the immune landscape, and these would differ from miRNAs in exosomes released from uninfected macrophages. As a means of investigating this hypothesis, we identified the profiles of miRNAs released by human macrophages that were either infected with *L. infantum* or uninfected. MDMs were derived from unrelated healthy human blood donors. Using the NanoString human miRNA panel, we observed that *L. infantum* infection caused MDMs to release significantly higher numbers of exosomes than uninfected MDMs, albeit similar in size. Furthermore, miRNA profiles of exosomes from infected MDMs were characterized by several miRNAs that were significantly up- or down-regulated compared to exosomes released by uninfected macrophages. Notably, there was a significantly higher amount of *hsa-miR-223-3p* in exosomes released from infected MDMs. This is of particular importance because *hsa-miR-223-3p* is involved in regulation of many infectious and inflammatory conditions (27).

Downmodulating *hsa-miR-223-3p* has previously been shown to increase the expression of IL-1β and IL-6 in macrophages (29). To further investigate a potential effect of increased miR-223-3p in human macrophages, we differentiated the human THP-1 cell line to assume macrophage-like characteristics and experimentally increased the intracellular *hsa-miR-223-3p* with a miR-mimic. The result was a significant decrease in NLRP3 in infected macrophages overexpressing *hsa-miR-223-3p*. We hypothesize that down-modulation of NLRP3 would inhibit inflammasome activation and potentially enhance parasite survival and growth in tissue-resident macrophages.

The current study is not the first to show a potential involvement of exosomes or miRNAs in leishmaniasis. Studies from our group and others have shown that exosomes are released from *L. infantum* promastigotes (11, 14, 37–39). We found that amastigotes release exosomes containing proteins that may aid in the parasite’s successful infection and survival in the host (Singh, Rodriguez, Wilson et al., under review). Host-derived exosomes have been implicated as modulators of the immune response in leishmaniasis (14, 37, 40). A few studies found evidence for individual miRNAs that might modify the immune pathogenesis of VL in dogs, mouse models, or *in vitro* cultured cells (27, 41, 42). These reported miRNAs did not include the miRNAs that were found to be up-regulated in exosomes from human MDMs, possibly reflecting the fact that the methods and model systems were different in each of these reports.

The most significant finding in this study of human exosomes was an increase on *hsa-miR-223-3p*. There is a precedent for an effect of either murine or human (*hsa*-) *miR-223-3p* on macrophage functions. Exosomes released from murine mesenchymal stem cells have been shown to influence nearby macrophages to shift toward an “M2” phenotype and promote skin wound healing, acting through murine *miR-223-3p* encapsulated in these exosomes (43). The exosomes released from primary human bone mesenchymal stem cells have also been shown to suppress the expression of inflammatory cytokines in THP-1 cells stimulated with LPS (17).

*miR223-3p* from human or murine sources is a myeloid-specific miRNA (44) that has been shown to skew macrophages to the anti-inflammatory phenotype (27, 28, 31, 32, 43, 45–47). *hsa-miR-223-3p* is also known to regulate the NF-κb pathway (45), a pathway that is important for initiating pro-inflammatory responses (48) and contributes to control of *Leishmania* spp. infections (49).

Consistent with our observations in this study of human cells, *miR-223-3p* has been shown to target and negatively regulate the expression of NLRP3 inflammasome in murine as well as human systems (30, 32, 45, 50). There are studies showing both a protective and a pathologic effect of NLRP3 activation in mouse models of leishmaniasis (51, 52). NLRP3 is activated in lesions of studies of humans with cutaneous leishmaniasis (53, 54), whereas in a study of murine macrophages. *L. donovani* was found to inhibit NLRP3 as a protective strategy against intracellular killing (51). Our findings are more consistent with a protective effect of NLRP3 against *L. infantum* infection of macrophages. Whether there are differences in the roles of NLRP3 cutaneous versus visceral leishmaniasis is an issue that remains to be resolved.

During the present study we selected a single transcript and showed it was modified by a single miRNA. However, miRNAs often have a subtle effect on individual transcripts which might not be detected experimentally, but they may have major effects on pathways since they can target several mRNAs in the pathway. Furthermore, up-regulated miRNAs can act synergistically and contribute to an overall modulation of a response pathway. It is likely that miR-223 has a wider effect on inflammasomes and inflammatory responses than we document here, as indicate by other studies (27).

In a previous study of a cohort of Indian patients with VL, our research group showed that *hsa-miR-223-3p* is increased in the circulating plasma exosomes compared to endemic controls (in press, Roy, Hudachek, Wilson et al.). This is consistent with the observation in the current study that *Leishmania* spp. infection of MDMs causes an increase in exosome-associated *hsa-miR-223-3p.* In the current study, we present evidence that overexpression of *hsa-miR-223-3p* by macrophages attenuates the parasite-induced expression of *NLRP3*. This effect could serve not only to aid parasite survival in tissue-resident macrophages in the spleen and liver, but also it could contribute to the overall immunosuppressive state seen in VL. There is a potential clinical role for inhibitors of miRNAs for treatment of humans. The use of miRNA inhibitors as potentially safe and effective therapeutics for infectious diseases (55–57) and cancer (58) is under investigation. Although it seems unlikely that an inhibitor of a human miRNA would provide specific and sufficient therapy for a parasitic disease, it is possible that inhibiting an miRNA that contributes to the pathogenic immune responses underlying leishmaniasis, such as *hsa-miR-223-3p*, might provide synergistic benefit to anti-parasitic therapy. In a setting where there are limited treatment options and parasites have become resistant to some existing treatments, there may be a role for combination therapy with an miRNA inhibitor. The above findings may have implications not only for the immune pathology, but also potential new approaches to treatment strategies for this parasitic disease.

## Data Availability

The original nanoString data presented in this study are found in the article's **Supplementary Files**. Inquiries about other studies can be directed to the corresponding author.

## Supplementary material

Supplementary Table 1 Full NanoString database. All results from the nanoString output are included. The samples included total RNA from uninfected (un) or infected human MDMs from each of three donors (1, 2, 3). Data represent the “counts” (arbitrary units) derived from fluorescence intensity of hybridization to each of 827 probes corresponding to the known human miRNAs and normalization controls. The data shown were normalized to the top 100 expressed miRNAs. The limit of detection was 20 counts, so rows in which all counts were 20 indicate miRNAs that were not expressed.

Supplementary Table 2 Predicted targeted transcripts of miR-223-3p and miR-1285. Predicted targets of hsa-miR-223-3p and hsa-miR-1285-5p were generated using miRDB and TargetScan. Targets predicted for both transcripts are shown in the final column of the table (column K). The Gene Ontology program available in the Panther list of classification programs (Version 18.0, https://www.pantherdb.org) was used to predict pathways from these lists. The targets found in the “Inflammation Mediated by Cytokine and Chemokine Signaling” for each miRNA are indicated, with the only two overlapping predicted targets highlighted in green.
